# Editorial: Microbial communities from geothermal fields: recent advances on characterization, interaction with the environment, and potential applications in biotechnology

**DOI:** 10.3389/fmicb.2023.1205759

**Published:** 2023-05-10

**Authors:** María Sofía Urbieta, Anna Panyushkina, Sara Cuadros-Orellana, Elena Gonzalez-Toril, Angeles Aguilera-Bazán, Jenny M. Blamey

**Affiliations:** ^1^CINDEFI (CONICET-UNLP), La Plata, Argentina; ^2^Winogradsky Institute of Microbiology, Research Centre “Fundamentals of Biotechnology” of the Russian Academy of Sciences, Moscow, Russia; ^3^Facultad de Ciencias Agrarias y Forestales, Universidad Católica de Maule, Talca, Chile; ^4^Centro Astrobiología (CSIC-INTA), Madrid, Spain; ^5^Biociencia Fundación Científica y Cultural, Ñuñoa, Chile

**Keywords:** environmental genomics, biogeochemistry, hydrogen metabolism, microbial community dynamics, CRISPS

Geothermal fields are unique habitats that provide a glimpse into the world of extreme environments and the fascinating microbial communities that inhabit them. These communities are known to exhibit wide-ranging adaptations to the harsh conditions they thrive in, high temperatures, low pH, and high concentrations of heavy metals, among others. Recent advances in microbial ecology and genomics have provided a wealth of information on the composition, structure, and function of these communities, revealing a complex web of interactions with their environment. Microbial communities in geothermal environments have caught the interest of the scientific community due to their potential applications in biotechnology, including bioremediation and the production of biofuels and biocatalysts; these applications have their basis in the metabolic and physiological adaptations to the extreme conditions. The articles included in this Research Topic use high-throughput sequencing, metagenomics, and bioinformatic and biostatistics tools to explore the microbial secrets of geothermal fields.

Power et al. made a substantial contribution to the understanding of temporal dynamics in geothermal microbial communities in Aotearoa, New Zealand by analyzing the 115 water column samples collected from 31 geothermal ecosystems over 34 months. The authors linked 16S rRNA gene high-throughput sequencing to numerous physicochemical parameters to ascertain microbial community stability and community response to both natural and anthropogenic disturbances and temporal variation in high temperature spring diversity across different pH values. The use of different statistical analysis improved the robustness of the conclusions compared to previous studies. At a broad scale, even though microbial communities may vary stochastically, drastic physicochemical change would be needed for drastic community changes. In fine-scale resolution, the results show that geothermal features with deeply sourced hydrothermal fluids provide steady environmental niches that stabilize microbial ecosystem structure.

With a similar approach Noell et al. studied the correlation between microbial community diversity and structure with geochemistry in the world's southernmost active volcano, Mt. Erebus in Antarctica, at two high temperature sites, one sheltered and other exposed to the harsh environmental conditions. Here, the use of cooccurrence network and biostatistics allowed finding significant correlations among the species present in each site. Within these significantly different environments, this work also shows that the microbial community is more affected by changes in pH than by variations in temperature.

To investigate how extremely acidophilic microbial species adapt not only to low pH conditions but also the high temperature and high salinity found in geothermal environments, Neira et al. performed a genome-guided prediction of the strategies used to cope particularly with low pH by strains of the extremely acidophilic methanotrophic genera *Methylacidiphilum, Methylacidimicrobium*, and Ca. *Methylacidithermus*, representatives of the deeply rooted phylum Verrucomicrobia. The predicted genes and pathways for acid resistance were organized in first and second line of defenses and allowed to infer a model of pH homeostasis mechanisms in these little studied genera. This comprehensive genomic analysis also shed light on the evolution of acid resistance in Verrucomicrobia, an ancient taxonomic group that could serve as a model to study the origin of life on Earth and other planets.

Along the same lines, Kucera et al. develop a model for the aerobic and anaerobic use of hydrogen in the acidophilic bacterium *Acidithiobacillus ferrooxidans*. Even though hydrogen is an available energy source in geothermal environments, hydrogen metabolism in acidophilic mesophilic bacteria is understudied. Here the authors investigate hydrogen metabolism and energy conservation associated with carbon assimilation from carbon dioxide, using state of the art multi-omics techniques, and explore the impact of these biological processes on the composition of the deep terrestrial subsurface.

Finally, Salgado et al. explored a topic rarely seen in assessments in geothermal environments, that is the phylogenomic diversity of Cas1 gene, a key component of the CRISPR-Cas system, which provides bacteria and archaea with adaptive immunity against invading genetic elements. A total of 2,150 Cas1 sequences were recovered from 48 metagenomes from different neutrophilic hot springs with a broad temperature range from three continents; the resulting ecological diversity of Cas1 and 16S rRNA was associated with geographic location. Several new Cas1 variants were discovered in geothermal microbial communities, suggesting that they may harbor an extensive reservoir of untapped genetic diversity.

In conclusion, the study of microbial communities from geothermal fields has provided valuable insights into the diversity, dynamics, and interactions of microorganisms in extreme environments. The scientific works discussed in this Research Topic have highlighted the importance of understanding the role of microbial communities in biogeochemical cycles and global biodiversity. The identification of novel genes, metabolic pathways, and acid resistance mechanisms in geothermal microorganisms have expanded our knowledge of microbial adaptation and evolution in extreme environments and open the door for future research on potential applications of these discoveries in biotechnology, including the development of biofuels, bioremediation, and bioprocessing.

Furthermore, the extreme chemical and physical conditions of geothermal environments that shape microbial community composition and diversity are providing a natural laboratory for the study of microbial ecology and evolution. The temporal dynamics of geothermal microbial communities underscore the importance of long-term monitoring and understanding of the environmental factors that affect microbial diversity and function.

## Author contributions

MU wrote and corrected the Editorial. All authors approved it for its publication.

